# Prediction of Thin-Walled Areas of Unruptured Cerebral Aneurysms through Comparison of Normalized Hemodynamic Parameters and Intraoperative Images

**DOI:** 10.1155/2018/3047181

**Published:** 2018-09-20

**Authors:** Kwang-Chun Cho, Ji Hun Choi, Je Hoon Oh, Yong Bae Kim

**Affiliations:** ^1^Department of Neurosurgery, College of Medicine, Catholic Kwandong University, International St. Mary's Hospital, Simgok-ro 100gil 25, Seo-gu, Incheon 22711, Republic of Korea; ^2^Department of Mechanical Engineering, Hanyang University, 55 Hanyangdaehak-ro, Sangrok-gu, Ansan, Gyeonggi-do 15588, Republic of Korea; ^3^Department of Neurosurgery, College of Medicine, Yonsei University, Gangnam Severance Hospital, 211 Eonju-ro, Gangnam-gu, Seoul 06273, Republic of Korea

## Abstract

**Object:**

Rupture of a cerebral aneurysm occurs mainly in a thin-walled area (TWA). Prediction of TWAs would help to assess the risk of rupture and select appropriate treatment strategy. There are several limitations of current prediction techniques for TWAs. To predict TWAs more accurately, HP should be normalized to minimize the influence of analysis conditions, and the effectiveness of normalized, combined hemodynamic parameters (CHPs) should be investigated with help of the quantitative color analysis of intraoperative images.

**Methods:**

A total of 21 unruptured cerebral aneurysms in 19 patients were analyzed. A normalized CHP was newly suggested as a weighted average of normalized wall shear stress (WSS) and normalized oscillatory shear index (OSI). Delta E from International Commission on Illumination was used to more objectively quantify color differences in intraoperative images.

**Results:**

CFD analysis results indicated that WSS and OSI were more predictive of TWAs than pressure (*P*<.001,* P*=.187,* P*=.970, respectively); these two parameters were selected to define the normalized CHP. The normalized CHP became more statistically significant (*P*<.001) as the weighting factor of normalized WSS increased and that of normalized OSI decreased. Locations with high CHP values corresponded well to those with high Delta E values (*P*<.001). Predicted TWAs based on the normalized CHP showed a relatively good agreement with intraoperative images (17 in 21 cases, 81.0%).

**Conclusion:**

100% weighting on the normalized WSS produced the most statistically significant result. The normalization scheme for WSS and OSI suggested in this work was validated using quantitative color analyses, rather than subjective judgments, of intraoperative images, and it might be clinically useful for predicting TWAs of unruptured cerebral aneurysms. The normalization scheme would also be integrated into further fluid-structure interaction analysis for more reliable estimation of the risk of aneurysm rupture.

## 1. Introduction

Spontaneous subarachnoid hemorrhages caused by ruptured cerebral aneurysms are fairly fatal and associated with high rates of morbidity and mortality. Cerebral aneurysm ruptures are likely to occur mainly in thin-walled areas (TWAs) within the aneurysms [1, 2]. Therefore, predicting TWAs is important for assessing the risk of aneurysm rupture and selecting the appropriate treatment.

TWAs have been predicted using computational fluid dynamics (CFD) with various hemodynamic parameters (HPs) [3–7]. Recently, the reliability of TWAs prediction has been improved by comparing CFD analysis results with intraoperative images of aneurysms [8–11]. However, there is still on-going controversy regarding which HP most accurately predicts TWAs. Meng et al. [3] reported that low wall shear stress (WSS) and high oscillatory shear index (OSI) triggered the growth and rupture of large aneurysm phenotypes, whereas high WSS was associated with the growth and rupture of small aneurysm phenotypes and medial thinning. Takao et al. [6] concluded that pressure loss coefficient might be a potential parameter to predict future rupture of unruptured aneurysms. Cebral et al. [12] analyzed a rupture risk of cerebral aneurysms considering flow pattern and impingement region.

There are several limitations of current TWA prediction methods. First, TWAs are predicted based on only single HP from CFD analysis although they are usually caused by complex phenomena [12–14]. Second, the value of HP from CFD analysis is greatly influenced by the inlet and outlet boundary conditions, which are not only hard to accurately obtain but also different from each patient. Third, identification of TWAs in intraoperative images is subjective and significantly dependent on the visual judgment of the clinician [9, 10, 15]. Finally, as aforementioned, it is still arguable which HP is the most appropriate to predict TWAs. To predict TWAs more accurately, the influence of CFD analysis conditions should be minimized by properly normalizing the HP value, and combination of normalized HP values could be introduced to enhance the prediction accuracy. The quantitative digital color analysis of intraoperative images would help to validate the effectiveness of normalized, combined hemodynamic parameters (CHPs).

In this study, we performed CFD analyses of 21 unruptured cerebral aneurysms and newly suggested both normalized schemes and a normalized CHP as a weighted average of normalized WSS and normalized OSI. We also investigated the influence of weighting factors of CHP on the prediction of TWAs. In order to avoid the clinicians' subjective assessment when determining the TWAs from corresponding intraoperative images, the quantitative digital color analyses were conducted based on CIE Delta E. The results of Delta E were compared with those of CHPs from CFD analyses to evaluate the effectiveness of the suggested normalization scheme and CHP for TWA prediction.

## 2. Materials and Methods

### 2.1. Patients and Image Data Acquisition

This study analyzed a total of 21 unruptured aneurysms in 19 patients (14 women and 5 men; mean age, 59 years; mean aneurysm size, 5.1 ± 1.15 mm). All data were obtained from patients treated via surgical neck clipping from July 2014 to December 2016 in our institution. The protocols used in this study were ethically approved by our institutional review board, and patient consent was waived. Ruptured aneurysms were excluded because it was difficult to obtain prior shape data and the shape was unclear. Digital subtraction angiography (DSA) images were generated using Artis zee biplane with syngo Workplace software (Siemens, Erlangen, Germany). All intraoperative images were captured using an OPMI Pentero surgical microscope (Carl Zeiss Microscopy GmbH, Jena, Germany) with a three-chip charge-coupled device digital video camera at 640 x 480 resolution, or a mounted Canon EOS 5D digital single lens reflex camera at 4368 x 2912 resolution.

### 2.2. Computational Fluid Dynamics

**Figure 1:**
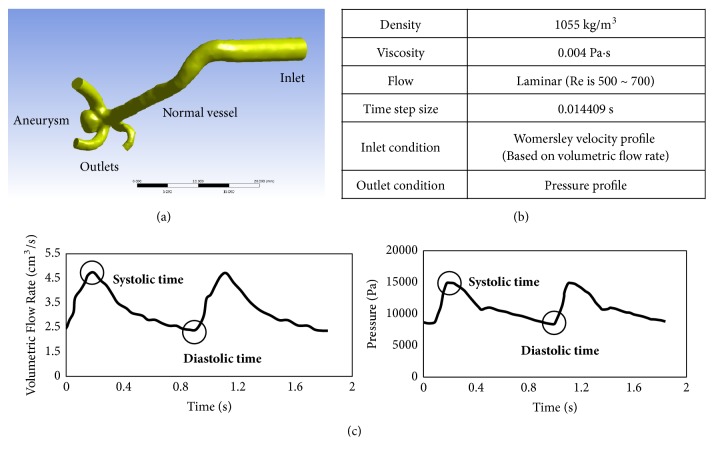
Detailed information on CFD simulation. (a) Example of CFD model with inlet and outlet long enough for reliable simulation. (b) Fluid properties and analysis conditions. (c) Flow rate profile for inlet condition and pressure profile for outlet condition.

Blood vessel and aneurysm geometry were generated from the DSA images. Blood vessel models were modified by using shape modification software (CATIA, V5-6R2012, Dassault Systems, Paris, France; Meshmixer, version 11.0.544, Autodesk, San Rafael, CA). Based on our previous study (Figure 1(a))[16], the inlet length of CFD models was set to 20 times larger than the inlet diameter, and the outlet length was set to twice larger than the outlet diameter to obtain reliable simulation results. CFD analysis was performed using ANSYS Fluent software (version 15.0, ANSYS Inc., Canonsburg, PA). The Navier-Stokes equations were solved to predict blood flow. Blood was modeled as an incompressible Newtonian fluid with a density of 1055 kg/m3 and a viscosity of 0.004 Pa·s, and the flow was assumed to be laminar. Blood vessels were modeled with the assumption of no-slip and rigid-wall conditions. Pulsatile flow with a Womersley velocity profile was used as the inlet boundary condition. Womersley velocity profile for each model was calculated from the volumetric flow rate of a human measured at the internal carotid artery [17]. Pressure profile, based on pressure waveforms measured at the superficial temporal artery [18], was applied at each outlet. Detailed information on CFD analysis is provided in Figures 1(b) and 1(c). After the mesh independence check, the number of meshes was determined to 4.5 million to 5.5 million.

### 2.3. Normalization Scheme and CHP

Individual HPs such as WSS, OSI, and pressure have different physical meanings, units, and value ranges, so direct comparison of their values is not reasonable. WSS is the dynamic friction force generated by viscous fluid moving along the surface of the solid wall and is defined as the product of fluid viscosity and shearing velocity gradient at the vascular wall [19]. OSI is a dimensionless parameter that indicates how the direction of WSS changes at a specific location during a cardiac cycle. In order to equally compare each HP's contribution, the value of HP should be normalized to have a value between 0 and 1.

4th-order equation based on the ellipse formula was proposed to create the normalized WSS,* WSS*_*norm*_, with a fixed range of 0 to 1 as follows:

(1) $$\begin{array}{c} {\left(\;WSS_{norm}-1\;\right)}^{4}+{\left(\;\frac{WSS-WSS_{max}}{WSS_{max}-WSS_{min}}\;\right)}^{4}=1 \end{array}$$

where* WSS*_*min*_ and* WSS*_*max*_ are the minimum and the maximum values of WSS in the aneurysm sac above its neck at the diastolic time, respectively. If* WSS* at the diastolic time is substituted into (1), then* WSS*_*norm*_ is calculated in the range of 0 to 1. Note that* WSS*_*norm*_ is 0 for* WSS*_*max*_ and 1 for* WSS*_*min*_.

*OSI* is originally defined as

(2) $$\begin{array}{c} OSI=\frac{1}{2}\left(\;1-\frac{\left|\int_{0}^{t}wss_{i}\;dt\right|}{\int_{0}^{t}\left|wss_{i}\right|dt}\;\right) \end{array}$$

where *wss*_i_ and* t* represent an instantaneous WSS vector and the cycle duration, respectively [20].* OSI* ranges from 0 to 0.5 and higher* OSI* values are more dangerous from a hemodynamic point of view; therefore,* OSI*_*norm*_ is also defined as a double of* OSI* to make its range become 0 to 1.

(3) $$\begin{array}{c} OSI_{norm}=2\times OSI \end{array}$$

Through this process, CHP can be defined as a weighted average of the* WSS*_*norm*_ and* OSI*_*norm*_ so that each HP contributes proportionately to the final CHP.

(4) CHP=w1×WSSnorm+w2×OSInorm

(5) w1+w2=1

where *w*_1_ and *w*_2_ are weighting factors for* WSS*_*norm*_ and* OSI*_*norm*_, respectively. By defining CHP as in (4), it also varies from 0 to 1.

### 2.4. Delta E

**Figure 2:**
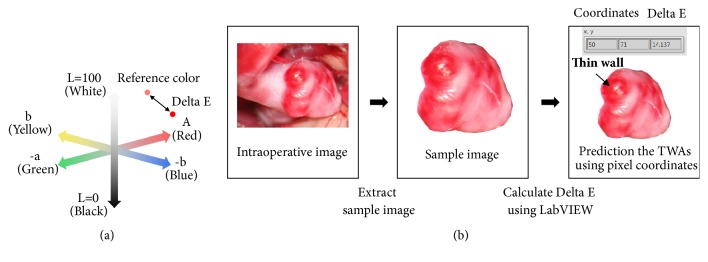
(a) CIE Delta E in the *L*^*∗*^*a*^*∗*^*b*^*∗*^ color coordinate system. (b) Procedure for calculating Delta E and predicting TWAs from an intraoperative image.

For more objective determination of TWAs, Delta E was introduced to quantify reddish areas on intraoperative images of patients' aneurysms. Delta E indicates how much a specific color differs from a reference color in CIE *L*^*∗*^*a*^*∗*^*b*^*∗*^ color coordinates. CIE *L*^*∗*^*a*^*∗*^*b*^*∗*^ is an internationally standardized colorimetric system for objective color expression, used in applications involving color recognition by the human eye. The color coordinates are composed of three axes: *L*^*∗*^ represents lightness and darkness, *a*^*∗*^ the degree of redness and greenness, and *b*^*∗*^ the degree of yellowness and blueness (Figure 2(a)).

To obtain Delta E, a region of interest was first extracted from an intraoperative image, then RGB values of all pixels of the extracted region were converted into *L*^*∗*^*a*^*∗*^*b*^*∗*^ values, and Delta E was calculated based on CIEDE 2000 color-difference formula[21] (Figure 2(b)). The obtained Delta E was finally modified to emphasize the lightness and redness of aneurysms as follows:

(6) $$\begin{array}{c} \Delta E_{m}=Delta\;\;E\cdot \sqrt{\frac{a_{2}^{2}}{L_{2}^{2}+a_{2}^{2}}} \end{array}$$

where *L*_2_ and *a*_2_ are the *L*^*∗*^ and *a*^*∗*^ values of specific area on the normal vessel selected by the clinicians. Δ*E*_*m*_ is the same as the projected Delta E along the *a*^*∗*^ axis. The most reddish area was identified with the highest value of Δ*E*_*m*_, and it was considered TWA. This process was performed using LabVIEW software (version 15.0, National Instruments, Austin, TX).

### 2.5. Statistical Analysis

Statistical analysis was conducted using SPSS software (version 21.0, IBM Corp, Armonk, NY). The Shapiro-Wilk test was used to evaluate the normality of parameters used. When satisfying the normality, statistical significance was analyzed using* t*-test. Otherwise, the Mann–Whitney* U* test was used for the statistical analysis. Statistical significance was assumed for values of* P*<.05.

## 3. Results

### 3.1. Usefulness of Delta E

**Figure 3:**
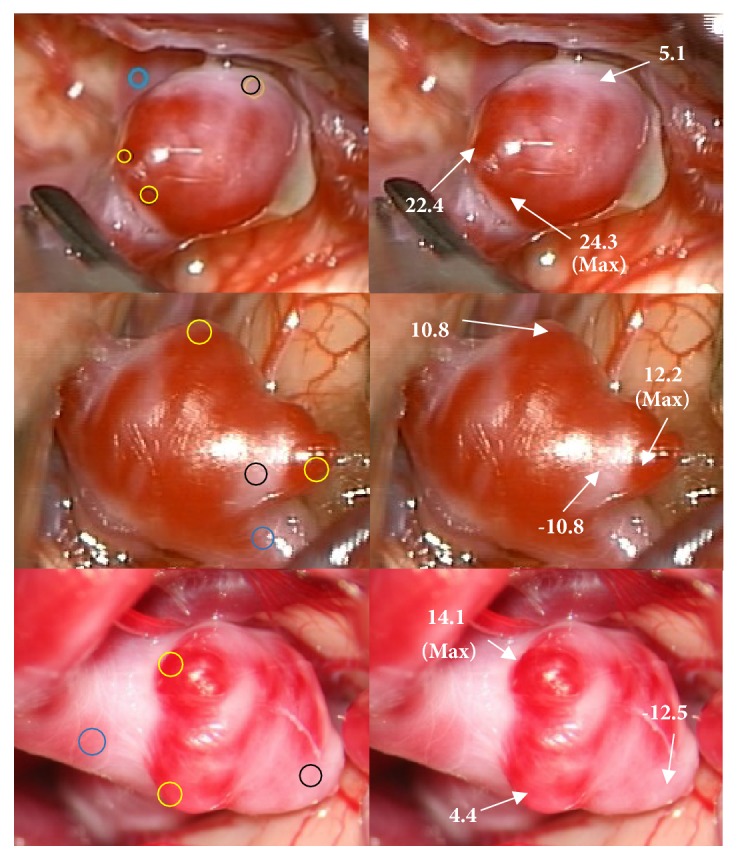
Demonstration of usefulness of modified Delta E, Δ*E*_*m*_ in intraoperative images. (Left) The blue circle is a reference region; the black and yellow circles are less dangerous and more dangerous regions, respectively. (Right) Calculated Δ*E*_*m*_ for selected regions relative the reference region.

To check the usefulness of Δ*E*_*m*_ in quantifying color differences of aneurysms in the intraoperative images, we first selected more dangerous, less dangerous, and reference regions and estimated Δ*E*_*m*_ for all other regions relative to the reference. Δ*E*_*m*_ results showed that the maximum value of Δ*E*_*m*_ was obtained at the most reddish area which was clinically regarded as more dangerous region (Figure 3). We also compared the values of Δ*E*_*m*_ between more dangerous and less dangerous areas for a total of 21 cases. Δ*E*_*m*_ of more dangerous areas was statistically higher than that of less dangerous ones (*P*<.001).

### 3.2. Representative Hemodynamic Parameters

**Figure 4:**
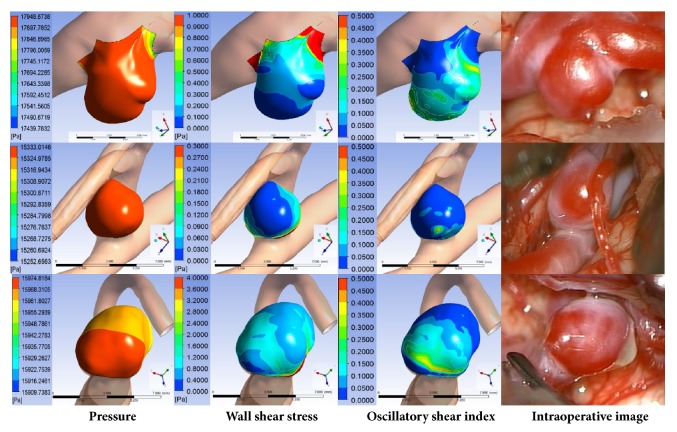
Comparison between the contours of HPs and intraoperative images.

It is still unclear which HPs would accurately predict TWAs [3, 6, 12]. Therefore, we analyzed the representative HPs, WSS, OSI, and pressure, using CFD analysis on 21 aneurysm cases. After the CFD models for 21 cases were visually aligned with the corresponding intraoperative images, each HP was obtained from the CFD results at positions corresponding to more dangerous (max. Δ*E*_*m*_) and less dangerous areas in the intraoperative images. WSS and OSI were more predictive HPs than pressure, based on statistical analysis (Table 1). Low WSS and high OSI corresponded to reddish areas in 17 and 7 cases, respectively, while pressure had nothing to do with reddish areas in the images (Figure 4).

### 3.3. Weighting Factors

Based on representative HPs results, we decided to include WSS and OSI but not pressure in constructing CHP and to give WSS more weight than OSI. To investigate the effect of weighting factors on CHP, statistical analysis was performed using a range of weighting factors, from 0.5 to 1 for* WSS*_*norm*_ (*w*_1_) and from 0.5 to 0 for* OSI*_*norm*_ (*w*_2_). For all 21 aneurysm cases, two CHPs were calculated from the same more dangerous (max. Δ*E*_*m*_) and less dangerous areas, respectively. As *w*_1_ was increased, the difference between the two CHPs increased and hence* P*-value decreased (Table 2). The less dangerous region showed an almost identical CHP value regardless of the combination of weighting factors.

### 3.4. Comparison of Estimated TWAs and Real TWAs

**Figure 5:**
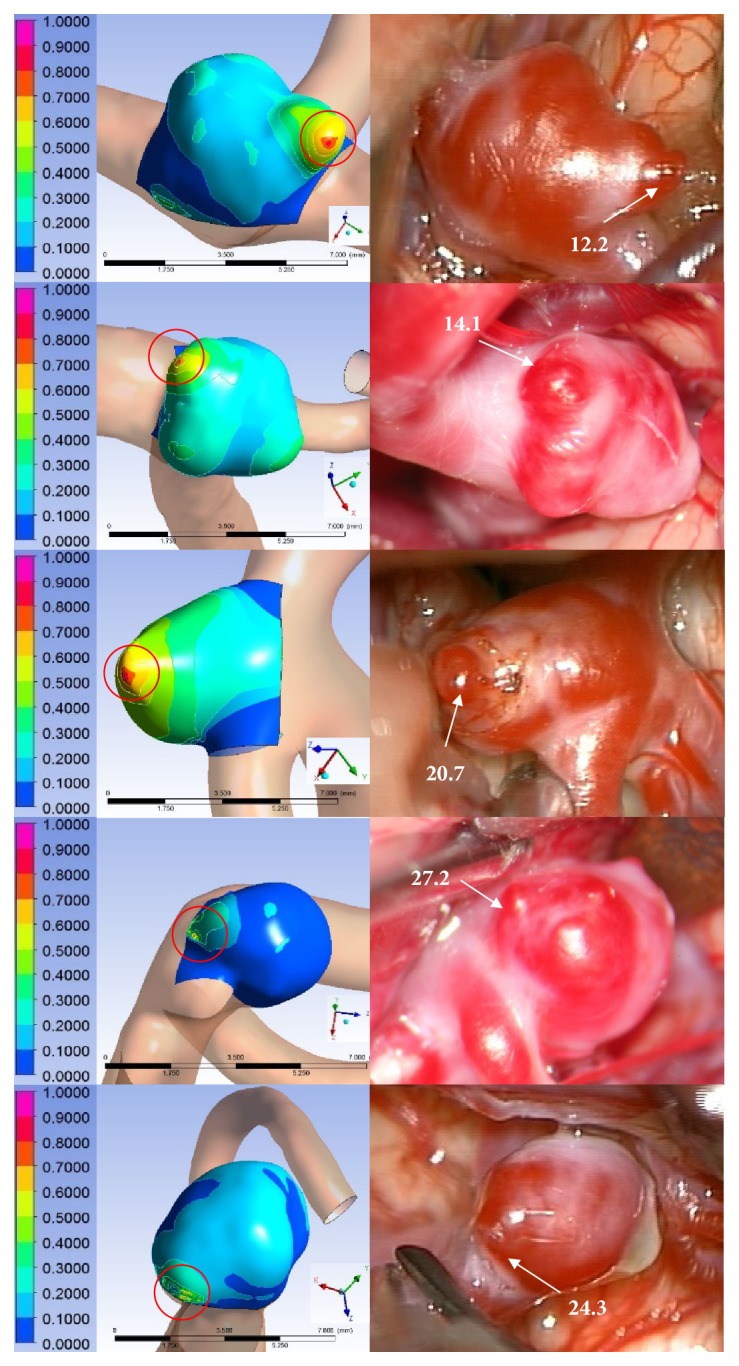
Representative correspondence cases. (Left) TWAs predicted using normalized CHP were compared with (Right) reddish areas of intraoperative images identified using Δ*E*_*m*_. The red circle is a TWA predicted using the normalized CHP.

**Figure 6:**
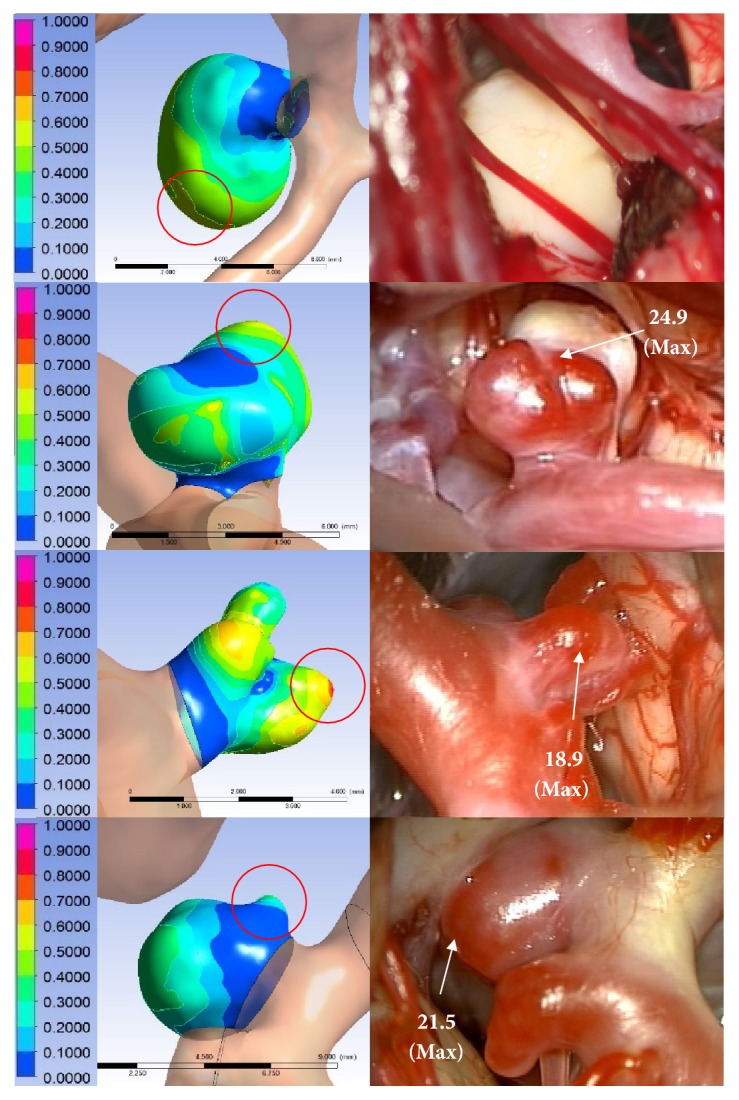
Representative noncorrespondence cases. Rows 1-2: severe atherosclerotic changes, row 3, influence of surrounding structure, or row 4: geometry differenc observed in the aneurysm.

For all 21 aneurysm cases, regions with the highest CHP values were compared with TWAs with max. Δ*E*_*m*_. Predictions of TWAs using the highest CHP matched in 17 of 21 cases (81 %), and regions with relatively high CHP values corresponded well to those with high Δ*E*_*m*_ values (*P*<.001, Figure 5). In the noncorrespondence cases, severe atherosclerotic changes, differences in geometry between CFD model and intraoperative image, and local influence of neighboring structure were observed in the aneurysms (Figure 6).

## 4. Discussion

This study is based on the idea that the aneurysm is mainly developed by hemodynamic mechanism. Histological studies of retrieved cerebral aneurysm walls have shown that a remodeling process of the aneurysm wall results in progressive wall thinning before aneurysm rupture. Although the rupture risk is dependent on several complicated factors as well as TWAs, we considered the TWA as the potential rupture region by focusing on the hemodynamic mechanism.

### 4.1. Delta E Analysis

In previous studies, TWAs were defined as reddish and transparent areas within aneurysms [9, 11, 22]. This definition relies on subjective visual judgment by clinical experts [9, 10, 15]. Kawaguchi et al. [10] reported that several clinical specialists, who did not know the CFD results before, compared intraoperative video at the same time and place to reduce human errors due to bias or subjective judgment. Suzuki et al. [9] compared CFD results with the clinicians' average scores on the redness of the aneurysms in intraoperative images to determine whether they were matched each other. However, these previous studies did not fully eliminate the limitations of subjective judgment.

Delta E is usually used for color quality control in industry. Since Delta E represents a relative difference (or distance) between two colors, it is suitable for determining the most reddish location relative to a reference location in a selected region of interest even when the region of interest is captured under a different light exposure or environment. Sometimes, two different colors show the same or similar value of Delta E relative to a reference color. This is because the two colors have almost the same distance from the reference color in the *L*^*∗*^*a*^*∗*^*b*^*∗*^ color coordinate system. If the line of Delta E is projected along the *a*^*∗*^ axis which represents the degree of redness, the component related to redness can be obtained from Delta E, leading to determining more reddish area. This was implemented using Δ*E*_*m*_.

### 4.2. Normalized CHP

From the analysis of 21 aneurysm cases, we found that low WSS and high OSI were more effective parameters for predicting TWAs, while pressure was not. These results are similar to those from previous studies. Xiang et al. [23] conducted CFD analysis using 38 ruptured and 81 unruptured aneurysms and showed that low WSS and high OSI were related to aneurysm rupture. Kulcsár et al. [24] reported from their CFD analysis that the pressure at aneurysm walls was relatively uniform.

Low WSS and high OSI cause upregulation of endothelial surface adhesion molecules, dysfunction of nitrous oxide-inducing flow, and increased endothelial permeability [18, 22, 25]. These phenomena can lead to weakening of the blood vessel wall, which may result in rupture [2, 26]. Weakening the blood vessel wall is hemodynamically complex phenomena, but little study has focused on the combination of HPs or its effect on the TWA prediction [12–14].

Before combining HPs, normalization of each HP should be done using a proper scheme. A range of 0 to 1 makes it easier to compare different HP contributions. Since WSS has both a maximum and a minimum value in an aneurysm and low WSS is related to TWA, a normalization scheme needs to be presented in such a way that the minimum WSS becomes 1 and the maximum WSS becomes 0. In order to give more weight on low WSS, we have proposed a 4th-order equation based on the ellipse formula as in (1). For OSI, it is already normalized from 0 to 0.5, and the value of 0.5 means more dangerous situation. Therefore, OSI is only doubled to match the value range from 0 to 1. Normalizing makes each HP with a different physical unit and value range contained between 0 and 1, so such normalization scheme also enables the value of each HP to be much less sensitive to inlet and outlet boundary conditions used in CFD analysis.

As listed in Table 2, the most significant difference between more dangerous and less dangerous areas was observed when *w*_1_ = 1 and *w*_2_ = 0, which means that* WSS*_*norm*_ only produced the best results. However, it should be noted that, for two out of 21 cases,* OSI*_*norm*_ showed better correlation with max. Δ*E*_*m*_ than* WSS*_*norm*_. In addition, almost identical CHP values regardless of weighting factors for the less dangerous region means that* WSS*_*norm*_ and* OSI*_*norm*_ have a similar value for that region due to the normalization effect.

### 4.3. Comparison of Normalized CHPs and Real TWAs

TWAs predicted using the highest CHP agreed with regions of max. Δ*E*_*m*_ in 17 of 21 cases while four cases did not match this trend. DSA is an imaging technique that only shows images of blood vessel lumen, so DSA image does not provide information on the thickness of blood vessel wall or any geometrical change inside the wall [27]. 3D aneurysm model obtained from DSA is not perfectly identical to the actual aneurysm shape observed in an intraoperative image. If an aneurysm has thick-walled areas due to atherosclerotic changes, the difference in shape becomes more severe. In some cases, the actual TWAs might be hidden or buried in the surrounding structures, so they could not be observed in the intraoperative images. As a result, a mismatch could occur in these cases.

### 4.4. Limitations

One of limitations of this study is that the boundary conditions in CFD analysis were not patient-specific. Although the influence of the boundary conditions is reduced by using the normalization scheme for HPs, application of patient-specific boundary conditions to CFD analysis is more desirable. Second, the number of cases investigated is relatively small. Additional data would allow for more accurate relationship between the normalized CHP and TWAs. Third, CFD analysis itself has technical limitations. Since blood vessels were assumed to be a non-slip and rigid wall for simplicity, it is difficult to determine whether rupture of aneurysm occurs from CFD analysis only. The elastic walls of blood vessels should be included in the analysis to investigate the rupture of aneurysms, which can be implemented by fluid-structure interaction (FSI) technique. Combination of FSI simulation and TWA prediction method suggested in this work would help to estimate the risk of individual aneurysm ruptures. This work is under way and will be addressed in future work.

## 5. Conclusions

We have suggested new, normalized schemes for WSS and OSI as well as the combination of the two as a weighted average of WSS_norm_ and OSI_norm_, which enable to predict TWAs of unruptured cerebral aneurysms from CFD analysis. Although there are several limitations, this study might provide a clinically potential way to predict vulnerability of cerebral aneurysm. It is expected that further FSI analysis that incorporates TWA prediction method proposed in this work would help to diagnose the future risk of target lesions and select the more appropriate treatment strategy for patients with cerebral aneurysms.

## Figures and Tables

**Table 1:** Comparison of estimated HPs (mean ± SD) at the more dangerous (max. *ΔE*_*m*_) and the less dangerous regions^a^.

HP	More dangerous region	Less dangerous region	P-value
Pressure, Pa	15755 ± 738	15752 ± 739	0.970
WSS, Pa	0.337 ± 0.705	1.759 ± 2.044	1.127E-04
OSI	0.073 ± 0.073	0.060 ± 0.084	0.187

^a^HP, hemodynamic parameter; OSI, oscillatory shear index; WSS, wall shear stress.

**Table 2:** Influence of weighting factors on CHP (mean ± SD) at the more dangerous (max. *ΔE*_*m*_) and the less dangerous regions^a^.

Weighting factor		CHP		P-value
*w*_1_	*w*_2_	More dangerous region	Less dangerous region	
0.5	0.5	0.359 ± 0.105	0.122 ± 0.124	2.669E-06
0.6	0.4	0.403 ± 0.108	0.122 ± 0.121	6.624E-07
0.7	0.3	0.449 ± 0.112	0.121 ± 0.118	1.517E-07
0.8	0.2	0.490 ± 0.121	0.121 ± 0.118	1.053E-07
0.9	0.1	0.534 ± 0.129	0.121 ± 0.120	9.342E-08
1.0	0.0	0.579 ± 0.140	0.121 ± 0.125	6.810E-08

^a^CHP, combined hemodynamic parameter.

## Data Availability

The data used to support the findings of this study are included within the article.
